# Patterns of feather mite (Acariformes: Astigmata) prevalence and load in a promiscuous bird during the breeding season

**DOI:** 10.1016/j.ijppaw.2024.101008

**Published:** 2024-10-20

**Authors:** Joanna Wołoszkiewicz, Jacek Dabert, Sandra Kaźmierczak, Janusz Kloskowski

**Affiliations:** a Institute of Zoology, Poznań University of Life Sciences, Wojska Polskiego 71C, 60-625, Poznań, Poland; b Department of Animal Morphology, Faculty of Biology, Adam Mickiewicz University, Uniwersytetu Poznańskiego 6, 61-614, Poznań, Poland

**Keywords:** *Acrocephalus*, Aquatic warbler, Astigmata, Ectoparasites, Promiscuity, *Trouessartia*

## Abstract

The effects of ectosymbiotic feather mites on avian host fitness and factors driving the variation in mite infestation levels, such as host mating and brood care system, are poorly understood. We investigated patterns of feather mite prevalence and abundance, and relationships between infestation and body condition in breeding Aquatic Warblers *Acrocephalus paludicola*, a promiscuous songbird with female-only parental care. In plumage, the dominant mite species was *Trouessartia bifurcata,* whose prevalence more than doubled during the breeding season, to reach 95% (95% confidence limits 71–99) during the second-brood period. Approximately 5% of birds were co-infested with *Proctophyllodes* cf. *clavatus*. While mite prevalence did not differ between sexes, mite abundance was significantly greater in Aquatic Warbler females than in males, and it increased between the first- and the second-brood period irrespective of sex. No significant relationship was found between mite prevalence or abundance and host condition expressed as mass scaled to size. However, in breeding females, this relationship could be confounded by the effects of nest-dwelling ectoparasites present in some nests but only sporadically if at all detected on adult birds; 2% of adult birds were heavily infested with the hematophagous mite *Dermanyssus hirundinis* (Mesostigmata). Our findings indicate that the mating system may affect symbiont variability between and within host species. The high prevalence of feather mites on adult birds at the late stage of the breeding season supports the hypothesis that promiscuous species have high infestation levels. Greater mite loads on Aquatic Warbler females than males reveal a different pattern from previously studied birds. However, we did not identify any consequences of mite infestation for the female body condition or current breeding success; hence, the apparently commensal relationship does not imply costs of female promiscuity.

## Highlights

• Host mating and care system may influence feather mite prevalence and abundance.
• Mite prevalence in adults of the promiscuous songbird Aquatic Warbler was high and increased over the breeding season.
• Females which care for their offspring alone hosted higher mite numbers than males.
• We found no relationship between infestation and female mass or fledgling production.

## Graphical abstract

**Figure:**
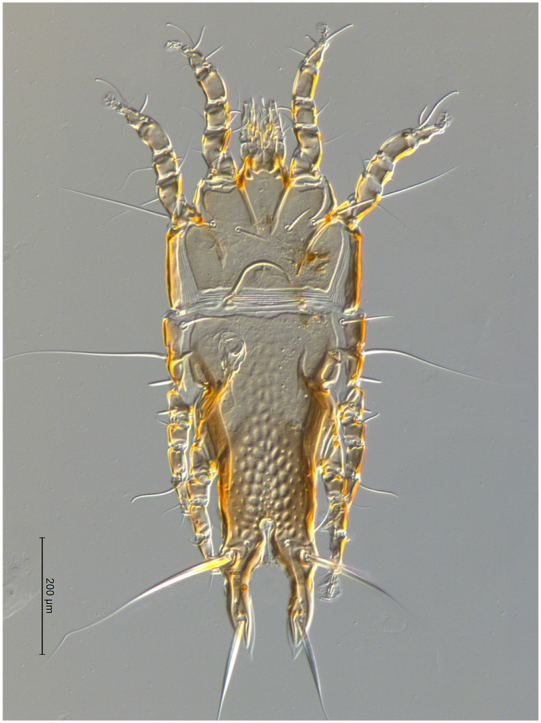

## 1 Introduction

The research on host-symbiont interactions has attracted the attention of evolutionary ecologists because of the costs and benefits conferred to hosts, whether and how hosts can control symbiont load, variation in prevalence and abundance of symbionts in host populations, and in symbiont transmission between hosts (e.g. Sapp, 1994; Poulin, 2011). Feather mites (Acariformes: Astigmata: Analgoidea and Pterolichoidea), symbiont arachnids which live on or in the feathers of birds, are dispersal-limited as they do not have a life history stage off the host (Proctor, 2003). Their transfer occurs mainly (though probably not exclusively) through direct contact between birds (Dubinin, 1951; Proctor, 2003). Their ambiguous relationships with avian hosts have been debated extensively. Though traditionally seen as parasitic (Atyeo and Gaud, 1979; Thompson et al., 1997; Harper, 1999; see also Galván and Sanz, 2006), recent studies suggest that, feeding on uropygial gland secretions, they are not measurably pathogenic, or they are even possibly beneficial to their hosts (Galván et al., 2012; Doña et al., 2019). An exception could be unnatural conditions, such as mass poultry farming, or when the host condition is reduced (Philips, 1993; Proctor, 2003). Many basic biological aspects of the symbioses, such as feather mite dispersal depending on the host specificity (Mironov and Malyshev, 2002; Matthews et al., 2022) and patterns of their prevalence and abundance within and between host populations (Diaz-Real et al., 2014; Matthews et al., 2018), including the phenological relationships between hosts and their mites (Blanco and Frías, 2001; Peet et al., 2022) are yet to be investigated. Feather mites transmit vertically from caring parents to nestlings (Proctor, 2003; Doña et al., 2017; Peet et al., 2022; but see Blanco et al., 1997), and the incidence of infestation and ectosymbiont abundance are likely to be greater in species with social behavior, e.g. group-living or commonly roosting (Blanco et al., 1997; but see Poiani, 1992; Figuerola, 2000). However, little is known about the influence of the host's mating and parental care system on the nature of the feather mite-bird relationship. Passing mites to nestlings may lead to a drop in their number in adults tending young (Pap et al., 2010); in previous studies on vertical transmission, if males were less involved in parental care, their mite load increased during the brood stage or the reduction in feather mite numbers was less pronounced than in females (Mironov, 2000; Doña et al., 2017). Feather mite load, which is positively affected by the activity (size) of the uropygial gland, whose productivity is stimulated by androgen levels, should be greater in breeding males (but see Pap et al., 2010). Although some studies have found higher loads on males (Blanco et al., 1999; Takagi, 2022), others failed to detect differences in feather mite abundance between sexes (e.g. Blanco and Frías, 2001; Dowling et al., 2001; Galván and Sanz, 2006; Davis and Cornelius, 2013).

The promiscuous mating and the associated intraspecific traits provide a yet unexplored evolutionary and ecological background for variation in the mite-bird symbioses. The horizontal transmission of ectosymbionts via sexual contacts should be facilitated compared to monogamous or even polygamous species. If certain ectosymbionts adversely affect the condition of their hosts, then contacts with multiple partners and also high frequency and duration of sexual activities affect infestation risk, and ectosymbionts may constrain female fitness benefits from multiple mating (Poiani and Wilks, 2000). The objective of this study was to examine the patterns of feather mite prevalence and abundance in breeding Aquatic Warblers *Acrocephalus paludicola* (Vieillot) (Aves: Acrocephalidae) and to investigate the association between infestation status and fitness components (body condition, breeding success) of the avian hosts. The Aquatic Warbler is a promiscuous songbird with uniparental female care (Dyrcz et al., 2002). Besides mating with multiple partners, an important feature of the mating system in the species is the extraordinarily long duration of copulations, with prolonged body contact between inseminations, presumably as a form of sperm competition and contact mate guarding (Schulze-Hagen et al., 1995). Moreover, a fraction of Aquatic Warbler females is double brooded (Dyrcz and Zdunek, 1993), which implies a long breeding season with a repeated cycle of mating. To our knowledge, this is the first study investigating patterns of infestation by feather mites in a bird species, in which both females and males mate with many partners. Insights into the potential effects of feather mites on Aquatic Warblers are of additional importance as the species is globally threatened due to habitat loss (Tanneberger and Kubacka, 2018). We compared the prevalence and abundance of feather mites between sexes and between the first-brood and the second-brood periods. We also related these infestation parameters to the body condition of the avian hosts and compared grooming behavior, as indicative of potential discomfort, between infested and non-infested nesting females. Owing to the promiscuous mating system, we predicted an overall high feather mite prevalence in the Aquatic Warbler, compared to previously studied non-promiscuous passerine species. If infestations occur during mating activities, we expected mite prevalence to increase between the early and late stages of the breeding season in both sexes, because birds involved in second broods would have, on average, more sexual mates with the progress of the season. Feather mite loads were expected to be higher in males, which had no opportunity to pass mites to nestlings.

## 2 Material and methods

### 2.1 Study species

The Aquatic Warbler is a small migratory passerine that breeds in open, mesotrophic lowland sedge fen mires and topographically similar marshy habitats created after fen drainage. Nests are placed on sedge tussocks or suspended in litter or living sedge vegetation (Kloskowski et al., 2015; Tanneberger and Kubacka, 2018). Though sexually monomorphic, the species exhibits a promiscuous mating system. Males do not defend territories, and all parental duties are performed by females (Dyrcz and Zdunek, 1993). Both males and females mate with multiple partners; female promiscuity results in intense male-male competition for insemination. Aquatic Warbler mating is characterized by numerous copulations, long duration of copulation mounting, and close physical contact for several minutes between inseminations (Schulze-Hagen et al., 1995). Most clutches are sired by multiple males, with up to five genetic fathers per brood (Dyrcz et al., 2002).

### 2.2 Study areas

Fieldwork was carried out in marshes and calcareous fen mires of Western Polesie Lowland, E Poland (51°16–22′ N, 23°25–54’ E). The breeding areas of the Aquatic Warbler were covered by Natura (2000) Special Protection Area “Chełm Calcareous Marshes” and the Polesie National Park, including a small marsh patch in the adjacent Ciesacin Reserve, SE Poland. The habitat was dominated to varying degrees by the sedge association *Cladietum marisci*, tall sedges *Magnocaricion,* and meadow grass associations, also locally invaded by reed *Phragmites australis*. The study area consists of scattered habitat patches, interspersed with agricultural land, stretching northeast for about 25 km.

### 2.3 Timing of breeding season

The Aquatic Warbler nesting season spanned from mid-May to mid-August and allowed for double-brooding. Based on observations of nesting females (and if data from nests of marked individuals did not indicate otherwise), we refer to the birds captured until the end of June as sampled during the first-brood period and those captured later in the season as sampled during the second-brood period. The division of the season was usually noticeable in the field by a time gap of 1–2 weeks in late June during which Aquatic Warblers were less active (Wołoszkiewicz et al., 2024; see also Dyrcz and Zdunek, 1993; Kubacka et al., 2014).

### 2.4 Field and laboratory procedures

Aquatic Warblers were captured using mist nets and sampled for mites during the breeding seasons of 2015–2017 and 2023. Males were attracted by tape luring near singing posts, while females were caught near their nests. We did not regularly examine nestlings; however, fledged young captured in mist nets alongside adult birds in July 2015–2016 were checked for feather mites. All Aquatic Warblers captured were ringed with individual combinations of aluminum and plastic color alphanumeric rings. Biometric measurements taken included tarsus length (using a caliper; to the nearest 0.1 mm), wing length from the bird wrist to the end of primary feathers (using a wing ruler), and weight (spring scale; to the nearest 0.01 g). Birds were aged by plumage as hatch-year or adults. The sex of adults was determined by the presence of a brood patch in females and of cloacal protuberance in males (Demongin, 2016). All birds were measured and inspected for mites in the field by the same researcher (JW) to avoid confounding effects due to differences between investigators.

Captured birds were checked for mite presence in the field by examination of the dorsal and ventral surfaces of each wing and tail feather aided by extension of both wings and the tail and their exposure to daylight. We also searched for mites by manually ruffling the body feathers. Mite presence was examined in good light conditions, not later than 20:00. Mites were detected only on the wings; in the infested birds they were found always on both wings, typically on innermost primaries and outermost secondaries, in more infested individuals spreading to other remiges and coverts. The abundance of feather mites was quantified. The time allocated to mite sampling was constrained by the requirement not to unduly disturb breeding birds, especially females, typically caring for clutches/broods at the time of capture. We used a visual examination method to classify mite abundance following Thompson et al. (1997) and Davis and Cornelius (2013), simplified to a four-point score: 0, no mites seen when the wing was extended; 1, occasional mites scattered among the primaries and secondaries, up to 30 mites per wing; 2, between 31 and about 100 mites present along the rachis of some primaries and secondaries, sporadically single mites on tertials and primary/greater coverts; 3, most remiges supporting many mites (hundreds on some primaries/secondaries), occasional mites on primary coverts, greater coverts, or both.

Whenever mites were detected, a visually representative sample was collected from the central part of the wing by careful snipping of a part (about 0.7 cm^2^) of a mite-harboring remige with small scissors. If mites were distinctly aggregated, two samples were snipped off from different wing areas. Innermost primaries and outermost secondaries were considered for sampling; however, occasionally greater coverts or outermost tertials were selected, and then whole feathers were collected (mean surface area 1.1 cm^2^, range 0.1–5.3 cm^2^ and one remige 12 cm^2^). The sampled feathers were individually stored in plastic string bags and kept frozen. After completing all the procedures the birds were immediately released at the site of capture. Dry mite individuals were softened in lactic acid (15%) for two days at room temperature and mounted on slides in Faure medium according to standard techniques for small mites (Krantz and Walter, 2009). Morphological analyses were performed with a Leica DM5500 B light microscope equipped with DIC illumination. Morphological species determination for feather mites (Astigmata) was carried out according to Atyeo and Braasch (1966) and Santana (1976), and for *Dermanyssus* (Mesostigmata) mites according to Moss (1968), Roy and Chauve (2007), and Mašán et al. (2014). The collected mites were counted under a stereomicroscope and their density was recalculated as abundance per 1 cm^2^ of the sampled feather(s). We recognize that both techniques of mite abundance estimation suffer from methodological issues. The subjective four-level scoring is an oversimplified index because of a broad range of mite numbers and as some species or their immature stages are too small to be readily counted in the field (including issues with distinguishing between live mites and skin casts). The quantitative assessment of mite loads based on the sampling of individual feathers or their parts is biased despite the apparent representativeness of the selected wing region because feather mites are not evenly distributed on the host body (Dabert, 1992; Mironov and Malyshev, 2002; Fernández-González et al., 2015; see also Labrador et al., 2021). Moreover, to determine the species/genus identity of mites present on a bird, in weakly or moderately infested individuals we targeted mite-infested feathers; such sampling is likely to overestimate the actual mite load. To correct for this bias, i.e., to sample from a larger, more representative area, for scarcely and unevenly infested birds (score 1 on the four-point scale) we recorded whenever the collected piece of the feather was a part of an otherwise non-infested feather or an even larger non-infested wing area. In such cases, we calculated mite density per whole feather or, in birds with a single infested feather per wing, per the total surface area of the analyzed feather and the next flight feather; surface areas were taken from measurements of the same feathers collected from other birds. This index can also underestimate the contribution of preimaginal stages which tend to hide under small and medium coverts and even use body covert feathers (Dabert, 1992). As the two measures of mite loads differed in the abundance estimation procedure, they were considered as complementary to one another (they were highly correlated, Spearman r = 0.906, n = 143, P < 0.001) and both were used in the statistical analyses. However, we acknowledge that they are only proxies of relative abundance, allowing for comparisons within the study population (given the same pattern of dominant mite species distribution among feathers), but poorly comparable with other studies on mite abundance in birds.

In 2015, nests were systematically searched for in the Chełm Calcareous Marshes. Areas were thoroughly searched where observations of Aquatic Warbler female behavior indicated the presence of a nest nearby (alarming, carrying food). Each nest position was recorded using a GPS (Garmin eTrex 20) and marked in a way not facilitating nest detection by predators. All active nests were visited at intervals of at least 3–4 days to determine the timing of nesting attempts and fledging success. To assess whether Aquatic Warblers respond to feather mite infestation by increasing grooming time, V8 (DTY/Shenzhen Industrial Co. Ltd) or GoPro Hero (GoPro, Inc.) mini-cameras were used. The camera lens was installed at the nest carefully, so as not to disturb the female. Battery capacity limited recording duration to about 5 h. Each focal nest was recorded at least once in the first and the second post-hatching week. The time that a female spent grooming (nearly exclusively preening with the bill) was recorded and the proportion of time dedicated to grooming was calculated. The recordings were obtained from a subsample of two contrasting groups of females, mite-infested (with large mite loads, the highest class of infestation) and mite-free females. We excluded nests where nest-dwelling parasite arthropods were recorded and could influence grooming behaviors; the eventual sample size was 13 nests (in total 125 h of observations).

### 2.5 Statistical analysis

We examined whether variation in the prevalence and load of feather mites could be attributed to the time of season and sex using Generalized Linear Mixed Models (GLMM). The GLMM analyses allowed us to control for non-independence of observations within years. A binomial GLMM with a logit link was used to explore the association between the probability of feather mite occurrence and time of season (first-brood vs second-brood period) and sex of the bird while a GLMM with a log link and Gaussian error distribution was applied to the abundance data (the approximated densities per feather surface area). The study year was incorporated as a random factor. As the mixed models consisted of a maximum of two predictor variables, inference was based on full models without applying predictor selection techniques; however, single-variable models produced the same conclusions. The significance of each variable was tested using Wald *χ*^2^ statistic (binomial GLMM) or F test (Gaussian GLMM). Predictions from the models were then back-transformed for reporting. We additionally analyzed the four-level scores of feather mite abundance using ordinal regression with a logit link function; all study years were pooled. Ordinal regression is a type of logistic regression that allows modelling of data classified into multiple levels with a natural ordering of the levels. The analyses were performed with GenStat 15 (VSN International Ltd).

Body mass corrected for structural body size was used to assess the individual condition of birds. To account for variation in body size, the scaled mass index (SMI), a measure of mass corrected for size, was calculated following Peig and Green (2009) using mass and wing length measurements. SMI was calculated separately for the sexes. We used wing length rather than tarsus length to calculate SMI because the former explained more variance in body mass (R^2^ = 0.134, df = 1, 139, P = 0.000008) than the latter (R^2^ = 0.112, df = 1, 136, P = 0.00006). We assessed the relationship between SMI of the examined Aquatic Warblers with feather mite occurrence and abundance using point-biserial and Spearman correlation, respectively, separately for each sex and for the first and second-brood periods.

Mite load analyses were based on the estimated relative densities of *Trouessartia bifurcata* (Trouessart) (Trouessartiidae) due to its predominance on Aquatic Warblers (see Results). A significance level of 0.05 was adopted. Each bird was used only once in the analysis. Sample sizes differed slightly between analyses because feather samples were lost or mass measurements were missing for a few birds with mites present.

## 3 Results

**Fig. 1:**
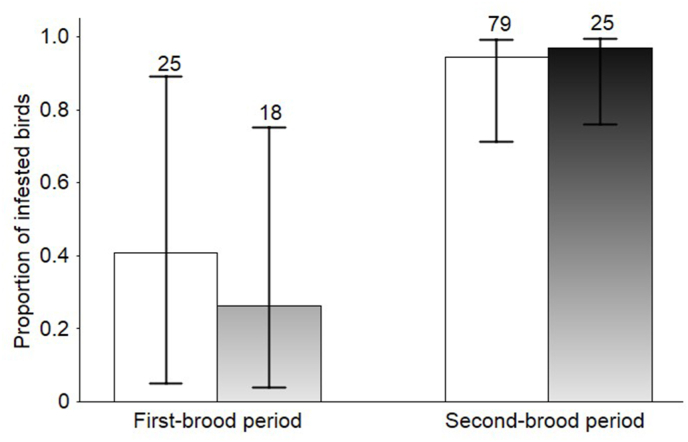
Back-transformed means and 95% CI from a binomial GLMM predicting the probability of feather mite presence on adult Aquatic Warblers during the first and the second-brood period. Males, open bars; females, filled bars. Sample size is given above each column.

**Fig. 2:**
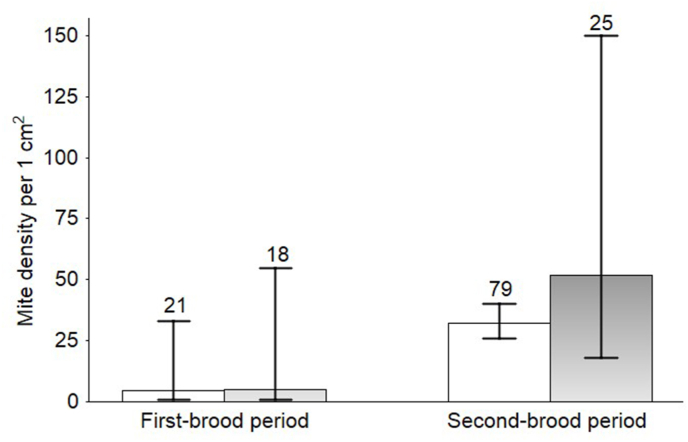
Feather mite abundance (back-transformed means and 95% CI) on the wing feathers of adult Aquatic Warblers during the first and the second-brood period. Males, open bars; females, filled bars. Sample size is given above each column.

We captured and sexed 147 adult Aquatic Warblers (104 males and 43 females). *Trouessartia bifurcata* was the dominant feather mite species. Feather mite prevalence did not differ between sexes (GLMM, Wald = 0.18, df = 1, P = 0.669), but more than doubled from the first to the second-brood period (Wald = 28.25, df = 1, P < 0.001; Fig. 1) reaching 95.2% (inverse logit back-transformed data, sexes pooled, 95% CI 71.0–99.4) during the second-brood period. Of 11 fledglings captured, mostly from the first broods, 7 were infested by *Trouessartia*, 5 of them heavily (score 3). All infested young birds carried adult mites; three fledglings had also larvae or nymphal instars. Feather mite load on adult birds was higher during the second-brood period (Wald = 4.46, df = 1, 144, P = 0.036) and females had significantly more mites than males (F = 8.30, 1, 144, P = 0.005; Fig. 2). The same conclusions could be drawn from the results of the ordinal regression; the scores for mite abundance increased from the first to the second brood period (*t* = 5.92, P < 0.001) and females were more infested than males (*t* = 2.04, P = 0.041). Of three late-captured (3–8 August) females in advanced molt (one examined for mites one day before the young left the nest, the other apparently not nesting at the time of examination), one has a low mite load (score 1), the two other were heavily infested (score 3).

The other feather mite recorded, *Proctophyllodes* cf. *clavatus* (Proctophyllodidae), occurred only on 7 birds (4.8%, always cohabiting with *Trouessartia*) at comparatively low densities (1.1–4.6 individuals per 1 cm^2^ of sampled feathers). *Trouessartia* typically occupied the dorsal and *Proctophyllodes* the ventral surface of remiges. Additionally, in mid-July, a mesostigmatid mite *Dermanyssus hirundinis* (Hermann), was detected on 2 females and 1 male (2.0% of sampled adults), all of which also hosted *Trouessartia*.

We found no correlation between SMI and feather mite infestation (presence/absence) or abundance for the total sample or when sexes were analyzed separately during both breeding stages (point-biserial or Spearman r; all P > 0.1). Infested and mite-free females did not differ in fledgling production (only the current broods of females were considered), Mann-Whitney U_15,12_ = 82, P = 0.714; median 3 (2–4 lower and upper quartile) and 4 (1.5–4.5), respectively. The proportion of time spent grooming did not differ between heavily infested and mite-free females, U_4,9_ = 12, P = 0.369, with low median values, 7.8 s/h (2.6–14.8) and 8.7 s/h (3.8–14.9), respectively.

## 4 Discussion

Our study reveals several key findings. Feather mite prevalence and abundance in breeding Aquatic Warblers increased between the first- and second-brood period. While mite prevalence did not differ between sexes, females hosted greater loads of mites than males. At the late stage of the breeding season, the prevalence of mites was high, supporting our prediction that promiscuous species should display high infestation levels, although high prevalence is also observed in some non-promiscuous birds (Peet et al., 2022). While our estimates of mite abundance are poorly comparable with studies in which other measures of abundance were used, they still point to relatively high mite loads on adult Aquatic Warblers (cf. Diaz-Real et al., 2014).

The within-season increase in mite loads suggests synchronization between the reproductive cycles of feather mites and the breeding stages of their hosts. Little is known about the phenology and development rates of individual mite species, but these traits are crucial for understanding the dynamics of symbioses with birds. Ectosymbionts likely adapt to track resource availability and synchronize their life cycles to maximize transmission opportunities (Blanco and Frías, 2001; Galván and Sanz, 2006; Marčanová and Janiga, 2021). Patterns of the seasonal changes in feather mite abundance appear to differ between species (Hamstra and Badyaev, 2009; Pap et al., 2010). The increase in feather mite abundance over the breeding season, possibly driven by mite reproduction, might also occur in birds with different mating systems (cf. Davis and Cornelius, 2013). However, this pattern is inconsistent with most previous studies on the within-season dynamics of mite infestation (Hamstra and Badyaev, 2009; Pap et al., 2010; Marčanová and Janiga, 2021; Peet et al., 2022). While a mite load increase was predicted in males, the hypothesis of a within-season reduction in the number of mites by females via a vertical transmission to nestlings (e.g. Mironov, 2000; Doña et al., 2017) was not substantiated, although the female is the single carer of the brood and thus is in frequent touch with chicks. Theoretically, adult mites can reproduce and remain on females until the feathers of the nestlings are fully developed (but see Hamstra and Badyaev 2009 on potential preference of mites for new feathers, including growing pinfeathers). In that case, mite abundance may be lower only in post-breeding females, no longer caring for young. However, mite load data from molting Aquatic Warbler females captured late in the season do not provide support for this concept. Our findings do not deny the importance of the vertical mite transfer, as most of the fledglings captured in our study were infested (note that they were mostly juveniles from the first broods, the period of relatively low mite prevalence in breeding females). However, regardless of the potential transmission to nestlings, and in the opposite direction of our prediction (cf. Davis and Cornelius, 2013; Hamstra and Badyaev, 2009), feather mite loads on breeding Aquatic Warbler females were overall higher than on males. These results should be treated with caution because of the poor proxies of mite load used; however, both indexes of mite abundance pointed to the same conclusions. As males vary in the number of sexual mates, some males may have fewer mating opportunities (thus having less opportunity to be infested) while presumably all females in our study were nesting individuals (i.e., all had mated). Therefore, one could expect lower total prevalence (but not abundance) of mites in males. However, this was not the case. Owing to parental effort, breeding females, especially when double brooded, with a short interval between first and second brood (Wołoszkiewicz et al., 2024), are under strong physiological stress and have less time for behavioral defense against mites, on the assumption that they form a burden to the host. On the other hand, it is not clear whether birds can effectively regulate feather mites during the breeding season, e.g. by grooming (Clayton, 1991; Dowling et al., 2001; Takagi, 2022). Finally, although feather mites are well adapted to resist airflow and feather friction during flight (Dabert and Mironov, 1999), the stages of avian incubation and brooding, as periods of reduced flight and lower exposure to sunlight, can favor reproductive activities and growth of mite infrapopulations.

Reports on the effects of feather mites on the condition of their avian hosts are unequivocal (Blanco et al., 1997, 1999; Harper, 1999; Bridge, 2003; Galván et al., 2012). We found no association between mite occurrence or abundance and body condition of adult Aquatic Warblers. This aligns with the recent view that feather mites are commensals or even mutualists, not feeding on vital bird resources (e.g. Pap et al., 2005; Matthews et al., 2018; Doña et al., 2019). However, other ectosymbionts might mask the effects of feather mites (cf. Hamstra and Badyaev, 2009; Matthews et al., 2018; for other issues with body condition metrics to assess costs/benefits of symbionts see Sánchez et al., 2018). In our study, ectoparasites present in nests, but often not recorded on birds captured outside the nest, were likely to induce physiological stress in nesting females. Evidently, the hematophagous *D. hirundinis* posed a greater danger to the fitness of breeding birds than astigmatid feather mites, as *Dermanyssus*, described as *D. gallinae*, has been reported to occasionally contribute to nestling losses (Vergeichik and Kozulin, 2006). We found this hematophagous species, novel for the Aquatic Warbler, on 2% of adult birds, but in nests it was present in a greater proportion (Wołoszkiewicz et al., 2024). Also, the fitness costs of mite infestation may be delayed in time. Previous research has not indicated any adverse effects of feather mite infestation on the annual survival of the hosts or reproductive performance (Pap et al., 2005; Matthews et al., 2018; but see Galván and Sanz, 2006). Similarly, we failed to obtain evidence that fledgling production by infested females was compromised. However, fledgling success in correlative studies is likely affected by factors independent of mite infestation, such as partial nest predation. If feather mite loads constitute a physiological burden to their avian hosts, the affected birds may simply reduce their reproductive investment in the current season (e.g. withdraw from the second breeding attempt) or in the next season. Such a response would be difficult to discern, as we captured advertising males or active females close to their nests. While our results add to the growing body of literature supporting the idea that feather mites are neutral to their hosts, the question remains whether they are not harmful when present in very large numbers (Haribal et al., 2005; Galván and Sanz, 2006). Aquatic Warbler females with large infestations did not groom more than non-infested females, indicating that feather mites did not cause behavioral discomfort or that grooming did not constitute an adequate defense (Dowling et al., 2001; see also Blanco et al., 1997). However, similarly to the argument above, grooming could be induced by nest-dwelling parasites; more importantly, the minute proportions of time devoted to grooming, as recorded by nest cameras, suggest that brood-caring females groom mainly outside the nest.

In conclusion, our data indicate that the breeding system of the avian host may influence the dynamics of feather mite infestation within the host population. Mite prevalence and loads increased with the breeding season's progress; although most of the Aquatic Warblers sampled early in the breeding season had likely already mated during the current spring, and so had an opportunity to be infested, both the incidence of infestation and abundance of feather mites were markedly lower than during the second brood period. Diaz-Real et al. (2014) have recommended controlling for the breeding season in research on mite intensity and prevalence due to pronounced seasonal variation; given the strong within-season increase in infestation levels observed in our study, we suggest accounting even for the stage of the breeding season. The low infestation levels of Aquatic Warblers at the early stage of the breeding season relative to the late part of the season indirectly indicate the ability of birds to dispose of the feather mites to some extent during the non-breeding period, presumably during molt (Takagi, 2022). Alternatively, annual cycles of mite infrapopulations are governed by intrinsic demographic rates, affected by perturbations due to unfavorable environmental conditions during migration or wintering. However, feather mites have developed adaptations to environmental stress, such as redistribution in response to feather molt or changes in ambient temperature (Dubinin, 1951; Wiles et al., 2000; Jovani and Serrano, 2001). The relatively high infestation level in Aquatic Warblers is concordant with the supposition that breeding adults of a promiscuous species are at significant risk of being infested by ectosymbionts. However, further research is needed to understand why females harbor more mites than males. Also, we found no evidence that infestation by feather mites or their abundance may function as a constraint of female promiscuity.

## CRediT authorship contribution statement

**Joanna Wołoszkiewicz:** Writing – review & editing, Writing – original draft, Resources, Project administration, Methodology, Investigation, Funding acquisition, Formal analysis, Conceptualization. **Jacek Dabert:** Writing – review & editing, Investigation, Data curation. **Sandra Kaźmierczak:** Investigation. **Janusz Kloskowski:** Writing – review & editing, Writing – original draft, Validation, Supervision, Methodology, Investigation, Formal analysis, Data curation, Conceptualization.

## Availability of data and materials

The datasets used and analyzed during this study are available from the corresponding author on reasonable request.

## Declaration of competing interest

None.
